# INTEREST GROUP SESSION—HUMAN-ANIMAL INTERACTION: PETS IN OLDER ADULTS’ SOCIAL NETWORKS: HUMAN-ANIMAL INTERACTION, INTERPERSONAL CONNECTIONS, AND HEALTHY AGING

**DOI:** 10.1093/geroni/igz038.724

**Published:** 2019-11-08

**Authors:** Jessica Bibbo, Sandy Branson, Jessica Bibbo

**Affiliations:** 1 Benjamin Rose Institute on Aging, Cleveland, Ohio, United States; 2 Cizik School of Nursing at UTHealth, Houston, Texas, United States

## Abstract

Empirical evidence supports positive associations between social support, interpersonal connections, and health as people age. This symposium addresses how human-animal interaction may facilitate connection throughout later life. Each talk presents unique ways pets: fit into social networks; expand interpersonal connections; and thereby, impact health and wellbeing. The first talk presents longitudinal associations of a history of pet ownership and marital status on health, in particular cognitive functioning, over time. The second talk presents qualitative evidence for how pets fit into older adults’ social network and quantitative evidence for the impact of animal and interpersonal companionship on overall and functional health. The third talk builds upon the literature linking dog walking with older adults’ physical health, by providing evidence for the positive impact of dog walking improving interpersonal connections with neighbors. The fourth talk discusses the influence of a unique intergenerational human-animal interaction service-learning course on university students’ attitudes towards older adults and those with disabilities. Enrolled students provide pet care (e.g., brushing, dog walking, delivering pet food and supplies) to low-income pet owners ages 60 and older and disabled adults. Students reported decreased biases towards older adults and those with disabilities after completing the course. The final talk is the first study to focus on the influence of pets in LGBTQ older adults living in rural southern Appalachia. Identifying as a LGBTQ person and living in a rural environment can present unique challenges and these qualitative results provide insight into pets’ influence on aging in this understudied population.

